# Expression of Sirtuins in the Retinal Neurons of Mice, Rats, and Humans

**DOI:** 10.3389/fnagi.2017.00366

**Published:** 2017-11-17

**Authors:** Hongdou Luo, Min Zhou, Kaibao Ji, Jiejie Zhuang, Wenjie Dang, Shiya Fu, Tao Sun, Xu Zhang

**Affiliations:** Jiangxi Provincial Key Laboratory for Ophthalmology, Affiliated Eye Hospital of Nanchang University, Nanchang, China

**Keywords:** retinal neuron, Sirtuins, aging, animal, human

## Abstract

Sirtuins are a class of histone deacetylases (HDACs) that have been shown to regulate a range of pathophysiological processes such as cellular aging, inflammation, metabolism, and cell proliferation. There are seven mammalian Sirtuins (SIRT1-7) that play important roles in stress response, aging, and neurodegenerative diseases. However, the location and function of Sirtuins in neurons are not well defined. This study assessed the retinal expression of Sirtuins in mice, rats, and humans and measured the expression of Sirtuins in aged and injured retinas. Expression of all 7 Sirtuins was confirmed by Western blot and Real-Time PCR analysis in all three species. SIRT1 is highly expressed in mouse, rat, and human retinas, whereas SIRT2-7 expression was relatively lower in human retinas. Immunofluorescence was also used to examine the expression and localization of Sirtuins in rat retinal neurons. Importantly, we demonstrate a marked reduction of SIRT1 expression in aged retinal neurons as well as retinas injured by acute ischemia-reperfusion. On the other hand, none of the other Sirtuins exhibit any significant age-related changes in expression except for SIRT5, which was significantly higher in the retinas of adults compared to both young and aged rats. Our work presents the first composite analysis of Sirtuins in the retinal neurons of mice, rats, and humans, and suggests that increasing the expression and activity of SIRT1 may be beneficial for the treatment of glaucoma and other age-related eye dysfunction.

## Introduction

The Sirtuins (SIRT) are an NAD+ dependent class III HDACs that share extensive homology with the yeast HDAC Silent Information Regulator 2 (Sir2) (Satoh et al., 2011). SIRT/Sir2 activity is crucial for lifespan extension by responding to metabolic and other environmental stresses. In mammals, seven Sir2 homologues (SIRT1-7) have been identified. They demonstrate primarily histone deacetylase (SIRT1, SIRT2, SIRT3, and SIRT5) or monoribosyltransferase activity (SIRT4 and SIRT6), which target histone and various non-histone proteins in distinct subcellular locations. SIRT1, SIRT6, and SIRT7 are predominantly in the nucleus (SIRT1 also has important cytoplasm functions). A SIRT1 fragment is present in the euchromatin, SIRT6 is present in the heterochromatin, and SIRT7 exists in the nucleolus, whereas SIRT2 is predominantly located in the cytoplasm and SIRT3, SIRT4, and SIRT5 are mitochondrial Sirtuins (Braidy et al., 2015).

The Sirtuins are involved in cell and tissue metabolism, making the study of Sirtuin expression in the retina important given that the retina is one of highest energy-consuming tissues in the body (Niven and Laughlin, 2008; Peng et al., 2010; Mimura et al., 2013). SIRT1 has been the best characterized of the seven mammalian Sirtuins (Morris, 2013). Recent studies have indicated that SIRT1 is involved in ocular aging, retinal neuron degeneration, and neuroprotection (Peng et al., 2010; Mimura et al., 2013). The retina generates ROS upon exposure to light (Sasaki et al., 2012), and Sirtuins may contribute to neuroprotection in the retina by regulating ROS. Higher expression of SIRT1 protects against diseases related to oxidative stress-induced ocular damage, such as cataracts (Zheng and Lu, 2011), age-related macular degeneration (Ozawa et al., 2010), and optic nerve degeneration in glaucoma patients (Mimura et al., 2013). Increased SIRT1 expression can be induced by neurotrophic factor signaling to protect retinal neurons and visual function (Zeng and Yang, 2015). Moreover, SIRT1 activators provide an important potential treatment to prevent permanent neurological dysfunction in optic neuritis and multiple sclerosis patients (Shindler et al., 2007). SIRT1 is also important in forestalling the effects of hypoxia-induced apoptosis in retinal ganglion cells (GCL) (Balaiya et al., 2012). SIRT3 may be a potential drug target candidate for eye diseases induced by retinal neovascularization (Mao et al., 2017). SIRT6 plays an important role in maintaining normal retinal function, likely by controlling histone acetylation levels and thereby regulating cellular metabolism and apoptosis (Silberman et al., 2014). In addition, SIRT6 has been implicated in retinal aging, and its deficiency in mice results in increased levels of retinal cell apoptosis (Shindler et al., 2007; Kim et al., 2010).

Sirtuins are expressed throughout the brain (hypothalamus), ears, muscle, liver, kidney, vasculature, skin, lungs, and other locations in the body (Morris, 2013). SIRT1 is expressed throughout the retina, including the retinal GCL, inner layer retinal cells (INL), photoreceptor cells, and retinal pigment epithelial cells (RPE) (Jaliffa et al., 2009). Another study found SIRT1 to also be expressed in the cornea, lens, iris, and ciliary body, in addition to the retina (Mimura et al., 2013). Very little, however, is known about the anatomical distribution and function of the other Sirtuins in the retina and other ocular tissues. To bridge this gap in knowledge, we designed a comprehensive analysis of Sirtuins and their expression in the retinas of multiple species. In this study, we characterized the mRNA and protein expression patterns of all the Sirtuins in normal adult mouse, rat, and human retinas, and measured the alterations of Sirtuin levels in aging and injured retinas.

## Materials and Methods

### Tissue Preparation

C57BL/6 male mice, 6–8 weeks of age; and Sprague-Dawley (SD) male rats, 2–3 months of age were obtained from Center of Laboratory Animal Science of Nanchang University. For aging study, the rats were used in the following age groups: young (2 months of age), adult (12 months of age), and old (20 months of age), which are roughly equivalent to human aged 20, 40, and 80 year old, respectively (Braidy et al., 2015). All procedures were approved by the Animal Care and Use Committees of Nanchang University Medical School and the ARVO statement for the Use of Animals in Ophthalmic and Vision Research. The animals were housed under standard conditions and were maintained in temperature-controlled rooms on a normal 12-h light/12-h dark cycle.

Six pairs of adult donor eyes (age range 30–50 years) were obtained from Red Cross Society of China Jiangxi Branch. All experiments involving human samples were approved by the Ethics Committee of Affiliated Eye Hospital of Nanchang University. All volunteer signed informed consent documents in written form in accordance with the principles of the Declaration of Helsinki. To obtain fresh eye tissues for RNA/protein and staining, we restricted the globes in the present study to those received less than 48 h postmortem. The previous medical and ocular histories of all donors were assessed to exclude donors with any eye disease. The retinas were processed as described below.

### Retina Neuronal Injured Model

Sprague-Dawley rats, 2–3 months of age were used. Under anesthesia (chloral hydrate), transient, unilateral, retinal ischemia was produced in rat eyes. Briefly, the anterior chambers of both eyes were cannulated and the IOP in one eye was elevated above systolic blood pressure (∼110 mm Hg) for 60 min and the contralateral eye was cannulated and maintained at normal IOP (Liu et al., 2006). Rats were killed at 0, 1, 3, and 7 days after retina ischemia.

### Real-Time PCR

Total RNA was extracted from each mouse, rat, and human retina using TRIZOL (Invitrogen) according to the manufacturer’s instructions. The concentration of isolated RNA was quantitated spectrophotometrically. Two hundred nanograms of total RNA from each sample was reverse transcribed into cDNA using the Quantscript RT Kit (TINGEN Biotech, Beijing, China). Specific primers for each gene (**Table 1**) were synthesized by TINGEN Biotech, Beijing, China. Real-time PCR was performed using the RealMasterMix (SYBR Green) kit (TINGEN Biotech, Beijing, China) with the following conditions: denature, 95°C, 20 s; annealing, 58°C, 20 s; and extension, 68°C, 30 s. All samples were tested in triplicate PCR reactions and the mean of the reactions was used for calculating the expression levels. All the data were collected from the linear range of each amplification. Expression levels were normalized to the average of tubulin mRNA levels from the same samples.

**Table 1:** Primers used in the study.

Primers	Mouse	Rat	Human
beta-actin	F-5′ CACTGTCGAGTCGCGTCC R-5′ CGCAGCGATATCGTCATCCA	F-5′ CCCGCGAGTACAACCTTCTT R-5′ CGACGAGCGCAGCGATA	F-5′ AAAGCCTGCCGGTGACTAAC R-5′ AGGAAAAGCATCACCCGGAG
sirt1	F-5′ CGGCTACCGAGGTCCATATAC R-5′ CAGCTCAGGTGGAGGAATTGT	F-5′ GGCACCGATCCTCGAACAAT R-5′ CGCTTTGGTGGTTCTGAAAGG	F-5′ TCTAACTGGAGCTGGGGTGT R-5′ TGGGAAGTCTACAGCAAGGC
sirt2	F-5′ GAGCCGGACCGATTCAGAC R-5′ AGACGCTCCTTTTGGGAACC	F-5′ TGAGCCTCAGACCCCTTTCT R-5′ AAGGGCAGACAGATACCCAC	F-5′ GCCCTTTACCAACATGGCTG R-5′ TTCGTACAACACCCAGAGCG
sirt3	F-5′ GGATTCGGATGGCGCTTGA R-5′ CACCTGTAACACTCCCGGAC	F-5′ AAGACATACGGGTGGAGCCT R-5′ GGACTCAGAGCAAAGGACCC	F-5′ AGAAGAGATGCGGGACCTTG R-5′ GGTCCATCAAGCCTAGAGCAG
sirt4	F-5′ GAGCATTCTTACTAGGGATTCCA R-5′ AACGGCTAAACAGTCGGGTT	F-5′ TACAGGTTCATCCTCACCGC R-5′ CAGGCAAGCCAAATCGTCAG	F-5′ GGCAGGAATCTCCACCGAAT R-5′ GCACTCCGGACAAAATCACC
sirt5	F-5′ GCCACCGACAGATTCAGGTT R-5′ CCACAGGGCGGTTAAGAAGT	F-5′ TTACCACTACCGGAGGGAGG R-5′ TGATGACCACAACCCGTCTG	F-5′ GGTGTTCCGACCTTCAGAGG R-5′ GTGGTAGAACTCCCACACCC
sirt6	F-5′ CCAAATCGTCAGGTCAGGGA R-5′ CAGAGTGGGGTACAGGGATG	F-5′ TGTCAACCTGCAACCCACAA R-5′ GGTGCTTCATGAGCTTGCAC	F-5′ AGTCTTCCAGTGTGGTGTTCC R-5′ TCCATGGTCCAGACTCCGT
sirt7	F-5′ CTAAGCGAAGCGGAGCCTAC R-5′ GTGGAGCCCATCACAGTTCT	F-5′ TGCAACACGTGGTGTCTCAG R-5′ GCAGGAGGTGCAGACTTCAAT	F-5′ GGTGGAGCGGGAATAGTCAG R-5′ CTGGGATAGACGCTGCACAT

### Western Blot Analysis

Retinal tissues were dissected from the retinal pigment epithelium and choroids and lysed in the RIPA buffer containing PMSF (Solarbio, China). The lysates were centrifuged, and supernatants were collected and subjected to western blot analysis. Protein concentrations were measured using the Bradford assay (Beyotime Institute of Biotechnology) according to the manufacturer’s instructions. Sample proteins (30 μg) were separated by 7.5–15% SDS-polyacrylamide gels and transferred to nitrocellulose membranes. The blots were blocked in 5% non-fat dry milk in Tris-buffered saline/Tween-20 and incubated with the appropriate primary antibodies (**Table 2**) followed by incubation with peroxidase-conjugated secondary antibodies (Cell Signaling). Finally, proteins on membranes were detected with enhanced chemiluminescence western blotting detection reagents (EMD Millipore).

**Table 2:** Primary antibodies used in the study.

Antibody	Source	Catalog no.	Type of Ab	Dilution	MW
SIRT1	CST	9475	Rabbit polyclonal	1:1000(WB) 1:100 (IHC)	120 kD
SIRT2	Sigma	S8447	Rabbit mAb	1:1000(WB) 1:100(IHC)	37 kD
SIRT3	CST	#2627	Rabbit mAb	1:1000(WB) 1:100 (IHC)	28 kD
SIRT3	Abcam	86671	Rabbit mAb	1:500(WB)	36.6 kD
SIRT4	Abcam	10140	Goat polyclonal	1:1000(WB) 1:100 (IHC)	36 kD
SIRT5	Abcam	78982	Rabbit polyclonal	1:1000(WB) 1:100 (IHC)	34 kD
SIRT6	CST	12486	Rabbit mAb	1:1000(WB) 1:100 (IHC)	42.36 kD
SIRT7	CST	5360	Rabbit mAb	1:1000(WB) 1:100 (IHC)	45 kD
Tubulin	TRANS	J10715	Donkey anti-mouse	1:1000(WB) 1:100 (IHC)	55 kD

### Immunohistochemistry

For immunohistochemical analysis, the animals were deeply anesthetized with chloral hydrate and then perfusion fixed through the left cardiac ventricle with ice-cold 4% paraformaldehyde (PFA) in 0.1 M phosphate saline buffer (PBS, pH7.4). After perfusion, eyes were enucleated and the anterior segments were removed. The posterior segments were fixed by immersion in 4% paraformaldehyde in 0.1 M phosphate buffer saline for 2 h. After washing in PBS, the tissues were transferred to 70% ethanol overnight, then dehydrated and embedded in paraffin. The 5-μm-thick paraffin tissue sections were dewaxed and then rehydrated. Endogenous peroxidase activity was blocked by placing the sections in 3% H_2_O_2_ in methanol for 30 min. For antigen retrieval, the sections were heated in 10mM sodium citrate buffer (pH 6.0) at a sub-boiling temperature for 10 min followed by cooling 30 min. Then the tissue sections were incubated with specific primary antibodies (**Table 2**) diluted in 5% bovine serum albumin (BSA) in PBS for overnight at 4°C. The sections incubated with PBS without primary antibody were used as negative controls. After several wash steps, the tissue sections were incubated with the secondary antibody, Alexa Fluor 594 donkey anti-rabbit IgG (H+L) for 1 h at room temperature. The sections were counterstained with DAPI (Vector Laboratories, Inc., Burlingame, CA, United States) and coverslipped. The staining was repeated three or more times for each antibody and the results were consistent.

### Data Analysis

All quantified data represented an average of at least three samples. SPSS 16.0 and Graph Pad Prism 5.0 software (Image J) were used for statistical analysis. All data are expressed as mean ± SD. Significance was established by paired *t*-test or Chi square test as appropriate. *P* < 0.05 was considered significant.

## Results

### Sirtuin Protein and mRNA Expression Levels in Normal Mouse, Rat, and Human Retinas

**FIGURE 1:**
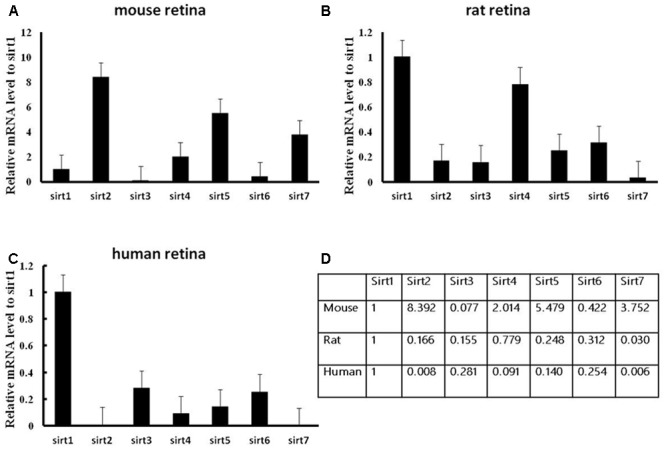
Expression of Sirtuin (SIRT1-7) genes in adult mouse, rat, and human retinal neurons. Histograms showing the SIRT1-7 mRNA levels in the retinas of mice **(A)**, rats **(B)**, and humans **(C)** as determined by qRT-PCR. The *y*-axis shows the Sirtuin expression levels relative to the SIRT1 gene. Data shown are based on the linear conversion of delta CT values for each sample (*n* = 5); error bars denote SEM. Statistical comparisons between the retinal expression levels of the different Sirtuins in each species are presented as a table **(D)**.

We firstly analyzed the mRNA levels of Sirtuins in mouse, rat, and human retinas by real-time PCR (**Figure 1**). The Sirtuins’ mRNA levels were different in each species. The mRNA level of SIRT1 was the highest in rat and human retinas, while SIRT2 was the highest in mouse retina. The mRNA level of SIRT7 was the lowest in rat and human retinas. SIRT3 mRNA was lowest in mouse retina. The mRNA levels of SIRT5, SIRT7, SIRT4, SIRT1, and SIRT6 were found in descending order in the mouse retina. Our results show that SIRT2, SIRT5, and SIRT7 were expressed primarily in mouse retina. Differences in the mRNA levels of SIRT2, SIRT3, and SIRT5 were not obvious in rat retina, while SIRT1, SIRT4, and SIRT6 were expressed at higher levels. The mRNA level of SIRT2 was almost as low as SIRT7 in human retina, while the mRNA levels of SIRT3, SIRT6, SIRT5, and SIRT4 descended in that order. We also discovered that SIRT1, SIRT3, and SIRT6 were primarily expressed in mouse retina (**Figures 1A–D**). Importantly, we confirmed that all seven Sirtuins were expressed in the retinas of all three species, and the high level of Sirtuin mRNA expression in the retina is consistent with the retina being one of the highest energy-consuming tissues in the body (Niven and Laughlin, 2008; Ban et al., 2013).

**FIGURE 2:**
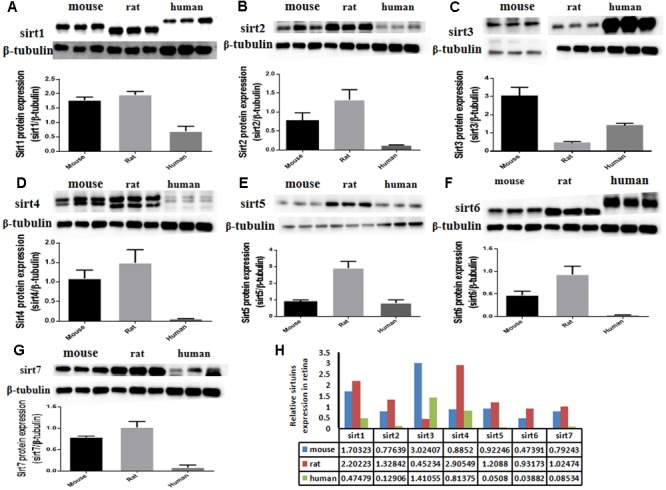
Sirtuin (SIRT1-7) protein levels in mouse, rat, and human retinal neurons. Representative western blots showing the protein levels for SIRT1 **(A)**, SIRT2 **(B)**, SIRT3 **(C)**, SIRT4 **(D)**, SIRT5 **(E)**, SIRT6 **(F)**, and SIRT7 **(G)** in the retinas of each species. The histograms in each panel show the densitometric mean and SEM normalized to the corresponding level of the loading control protein β-Tubulin (*n* = 4 independent subjects). Statistical comparisons between the expression levels of the different Sirtuin proteins in the retinas of adult mouse, rat, and human are presented as a table **(H)**.

**FIGURE 3:**
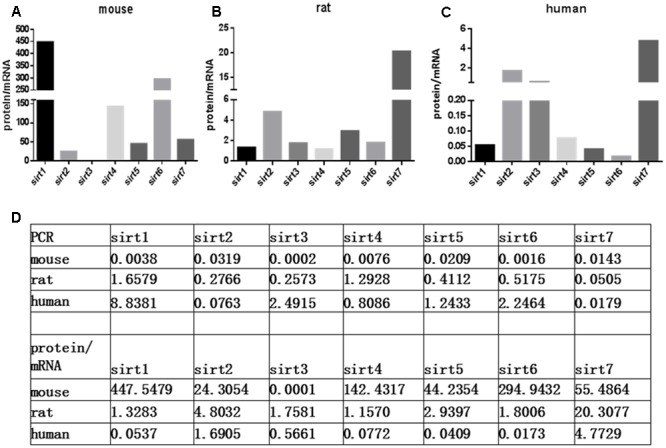
Relative Sirtuin protein to mRNA ratios in mouse, rat, and human retinal neurons. The ratios of protein levels from western blot to mRNA levels from Real-Time PCR are denoted on the *Y*-axis for each Sirtuin family member (*X*-axis) in the retinas of mice **(A)**, rats **(B)**, and humans **(C)**. Supplemental data about PCR and protein/mRNA are displayed **(D)**.

The protein expression levels of Sirt1 and Sirt2 were highest in the retina of rats, while their levels were lowest in human retina (**Figures 2A,B**). Sirt3 protein was detected in mouse retina, and was observed at a low level in rat retina (**Figure 2C**). Expression of Sirt4 and Sirt5 in human retina was not obvious whereas their expression was more visible in mouse retina than in rat (**Figures 2D,E**). Sirt6 and Sirt7 were expressed in mouse, rat, and human retina, and were lowest in human retina (**Figures 2F,G**). Our results demonstrate that the protein levels of the Sirtuins in mouse retinas descend in the following order: SIRT3, SIRT1, SIRT5, SIRT4, SIRT7, SIRT2, and SIRT6 (**Figure 2H**). The protein levels of the Sirtuins in rat retinas descend in the following order: SIRT4, SIRT1, SIRT2, SIRT5, SIRT7, SIRT6, and SIRT3. Finally, the protein levels of the Sirtuins in human retinas descend in the following order: SIRT3, SIRT4, SIRT1, SIRT2, SIRT7, SIRT5, and SIRT6 (**Figure 2H**). Remarkably, we found the protein levels of the Sirtuins, including that of SIRT1 and SIRT3, but especially SIRT5 and SIRT6, in human retina did not correspond to their respective mRNA levels (**Figure 3**).

### Expression and Cellular Localization of Sirtuins in Normal Mouse, Rat and Human Retinas

**FIGURE 4:**
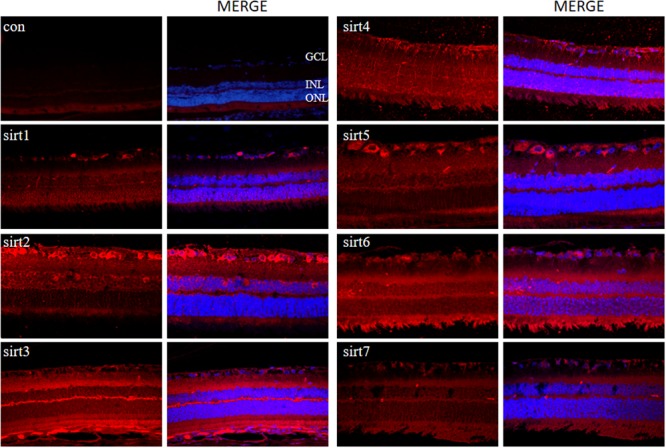
Immunolocalization of Sirtuin proteins in retinal cross-sections of rat. Retinal cross sections prepared from rat eyes were immunostained with Sirtuin antibodies. The results are displayed as negative control (without primary antibody), SIRT1, SIRT2, SIRT3, SIRT4, SIRT5, SIRT6, and SIRT7 in adult rat retinas. All photographs were taken at 40× magnification. Con, control; GCL, ganglion cell layer; INL, inner nuclear layer; ONL, outer nuclear layer.

Since all Sirtuins were expressed in rat retina, we selected normal adult rat retina for immunofluorescence detection. We observed that SIRT1, SIRT 6, SIRT7 were localized in the nucleus, SIRT2 was predominantly in the cytoplasm, SIRT3, SIRT4, SIRT5 were localized in the mitochondria (**Figure 4**). SIRT1 expressed in the GCL layer, inner nuclear layer, outer nuclear layer and retinal pigment epithelium. SIRT2 mainly expressed in the GCL layer, outer plexiform layer and retinal pigment epithelium. Meanwhile, SIRT3 mainly expressed in the inner plexiform layer, outer plexiform layer and retinal pigment epithelium, but was less expressed in the GCL layer. SIRT4 mainly expressed in the GCL layer and inner nuclear layer in the form of threadiness. SIRT5, SIRT6 and SIRT7 mainly expressed in the GCL layer, inner plexiform layer and retinal pigment epithelium. From the overall analysis, all Sirtuins except SIRT3 were expressed in the GCL layer, while all Sirtuins except Sirt4 were expressed in the retinal pigment epithelium.

### Decreased Sirt1 Levels in Aging Retinas and Injured Retinas

**FIGURE 5:**
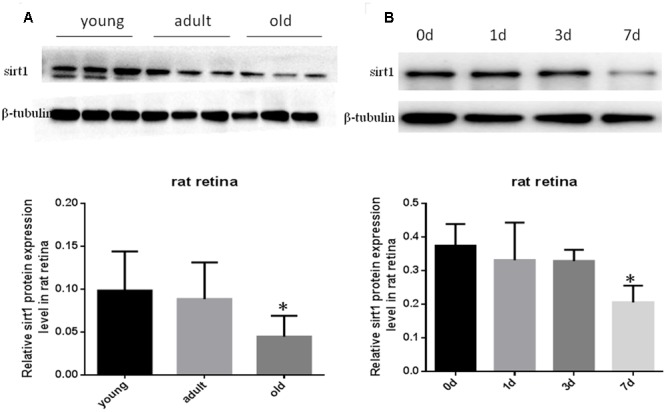
Protein levels of SIRT1 in aging and injured rat retinas. Representative Western blot showing SIRT1 protein levels in aged (old) retinas compared to young and adult retinas **(A)**. SIRT1 protein levels in rat retinas following transient retinal ischemia-reperfusion injury **(B)**. Densitometry analysis of SIRT1 in aged and injured retinas and normalized to β-tubulin is shown below the representative blots (^∗^*P* < 0.05, *n* = 4).

**FIGURE 6:**
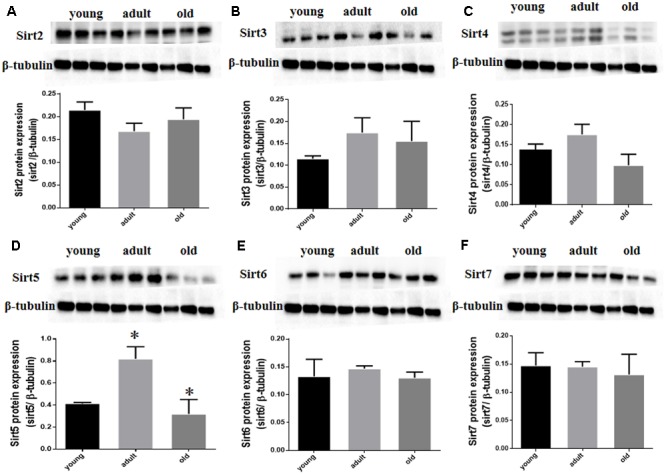
SIRT2-7 protein levels in aging rat retinal neurons. Representative western blots showing the protein levels for SIRT2 **(A)**, SIRT3 **(B)**, SIRT4 **(C)**, SIRT5 **(D)**, SIRT6 **(E)** and SIRT7 **(F)** in the retinas of young, adult, and old rats. The histograms in each panel show the densitometric mean and SEM normalized to the corresponding level of the loading control protein β-Tubulin (^∗^*P* < 0.05, *n* = 4 independent subjects).

Prior studies have suggested that SIRT1 is involved in retinal neuron degeneration during aging (Zeng and Yang, 2015) and retinal injury (Alcendor et al., 2007; Mortuza et al., 2015). We therefore analyzed SIRT1 expression in rat retinas at different ages as well as before and after retinal injury. Compared to young and adult retinas, the aged (old) retinas exhibited lower SIRT1 protein levels (**Figure 5A**). SIRT1 protein levels also decreased in the retinas following acute retinal ischemic injury (**Figure 5B**). On the other hand, SIRT3-5 (mitochondrial associated Sirtuins) were all expressed at the highest level in adults but were not significantly lower in old rats (**Figures 6B–D**), while the expression levels of SIRT2, SIRT6, and SIRT7 did not significantly change with age (**Figures 6A,E,F**).

## Discussion

To date, the significance of Sirtuin expression in the retina is unknown. To establish a basis for studying the pleiotropic functions of the Sirtuins in the retina, we analyzed the expression pattern and location of the Sirtuins (SIRT1-7) in the aging retinas of different species at the mRNA and protein level. Our results demonstrated that all members of the Sirtuin family are detected in the retinal neurons of mouse, rat, and human, which is in agreement with previous observations that, in the mice retina, certain cells express SIRT1 (Geng et al., 2016). Moreover, our study shows that SIRT1 expression is decreased in aged retinas as well as ischemia-reperfusion injured retinas.

In the present study, we demonstrated that certain Sirtuins are abundantly expressed in the retinas of mouse, rat, and human, and there are some similarities in the expression patterns of Sirtuins among different species. However, there is also considerable variability in the localization and distribution between Sirtuin family members. We found that the mRNA and protein content of SIRT1, which is the earliest discovered and most studied member of the Sirtuin family, is abundant in mouse, rat and human retinas. In previous studies, SIRT1 (Zeng and Yang, 2015) and SIRT6 (Silberman et al., 2014) could both protect normal retinal function, likely by controlling histone acetylation levels and thereby regulating cellular metabolism and apoptosis, but the exact mechanisms remain unknown.

Interestingly, SIRT3 protein was not only detected in mouse, rat, and human retinas, but was highly expressed in human retinas with different antibodies. Overexpression of SIRT3 increases mitochondrial respiration and reduces reactive oxygen species (ROS) production, which may explain its high expression in human retina in order to protect the organ from oxidative stress-induced injury (Shulyakova et al., 2014). Notable discordance between the mRNA and protein expression patterns was also observed for SIRT1, SIRT3, SIRT5, and SIRT6 in human retinas. This suggests that post-transcriptional mechanisms contribute to the regulation of protein expression of Sirtuins, possibly via RNA-binding proteins (RBPs) or non-coding small RNAs (Sidorova-Darmos et al., 2014). Interestingly, the Sirtuin expression levels we observed in this study in rat retinas, in which Sirt1 had very high expression and others had abundant and well-proportioned expression, were significantly different from the patterns observed in rat brain, where SIRT2, SIRT3, and SIRT5 are abundant but others showed lower levels (Sidorova-Darmos et al., 2014). Further investigation of specific Sirtuin family members, their unique expression patterns, and regulatory mechanisms during development, injury, survival, and rejuvenation will be valuable for the retina as well as other tissues.

In the retinas of mice, rats, and humans, SIRT1 can be observed in the GCL, INL, ONL and RPE, whereas other studies have reported that SIRT1 is only expressed in the GCL, INL, and RPE of rat retinas (Kim et al., 2013) or in the GCL, INL, and ONL of mouse retinas (Jaliffa et al., 2009). In general, SIRT1 was expressed in retinal tertiary neurons, which are in close contact with retinal neurons and serve a protective function. Given our finding that retinal SIRT1 is decreased by aging and injury and the previous finding that SIRT1 can protect against optic nerve degeneration in glaucoma patients (Mimura et al., 2013), it is possible that SIRT1-activating drugs such as resveratrol may prevent or treat traumatic optic nerve injury as well as various optic neuropathies that involve oxidative stress (Zuo et al., 2013). Moreover, our study provides evidence for SIRT1 activators in the treatment of glaucoma.

We found that SIRT3 is mainly in the GCL, IPL, OPL, and RPE of rat retinas. Previous reports have demonstrated that SIRT3 is present in the mitochondrial matrix and can regulate mitochondrial function (Someya et al., 2010). Further investigation may be necessary to delineate the differences in SIRT3 distribution between human and mouse retinas, and whether SIRT3 may offer direct protection for the GCLs. SIRT4, another mitochondrial protein that regulates energy metabolism (Ho et al., 2013), is primarily expressed in the GCL, INL, and ONL in a thread-like pattern that may be closely associated with astrocyte cytoplasm. One prior study showed that Sirt4 is highly expressed in astrocytes in the postnatal brain and in radial glia during embryogenesis (Komlos et al., 2013), where activation of SIRT4 may reflect a regulatory function in retinal gangling cells through activating/restraining astrocytes. We also found that SIRT6 was expressed in the GCL, IPL, and RPE in rat retinas. Interestingly, recent studies have uncovered novel physiological functions of SIRT6, including acting as a mono-ADP-ribosyltransferase for PARP1 under oxidative stress and stimulating PARP1 function to support DNA repair (Mao et al., 2011). Considering the protective effects of SIRT6 to retina function (Silberman et al., 2014) and retinal aging (Shindler et al., 2007; Kim et al., 2010), further investigation of the post-transcriptional regulation of Sirt6 expression and its role in retinal disease is needed. In the pathogenesis of glaucoma, RGC apoptosis occurs. As all Sirtuins are expressed in the GCL, we hypothesize that dysfunction of Sirtuins may be closely related to the pathogenesis of glaucoma, and that Sirtuins might become a new target for glaucoma treatment.

Previous studies have shown that SIRT1 plays a crucial role in age-related retinal degeneration (Ozawa et al., 2010; Pennington and DeAngelis, 2016). Its activity has been studied in various animal models of neurodegenerative diseases, including Huntington’s disease (Jeong et al., 2012), Alzheimer’s disease (Kim et al., 2007), and retinal ischemic injury (Duan, 2013). In the present study, we examined the expression of SIRT1 in aged and injured retinas and detected decreased SIRT1 expression in retinal neurons following aging and ischemic retina injury. Aging is a known risk factor for many neurodegenerative diseases including Alzheimer’s disease, Parkinson’s disease (Wu et al., 2011), Huntington’s disease, and glaucoma. Studies have demonstrated that SIRT1 is responsible for the protective effects of resveratrol, a SIRT1 activator, against neurodegeneration and aging (Peng et al., 2010). We provide observational support for a role for SIRT1 in the protection of retinal neurons from aging and ischemic injury. Further investigation is needed to confirm that boosting SIRT1 expression and activity will offer protection against retinal neurodegeneration. Interestingly, we observed two SIRT1 bands in young rat retinas but not adult or aged retinas, which may be due to the presence of a second isoform (splicing or post-translational cleavage) or to post-translational modifications (phosphorylation, ubiquitin, etc). According to Uniprot there are two known isoforms for human and mouse SIRT1, so it is possible there are two isoforms in rats as well. Moreover, SIRT1 is known to be highly regulated by post-translational modifications including phosphorylation, methylation, and *S*-nitrosocysteine, and therefore PTMs could also be responsible for the two SIRT1 bands seen in young adults. It is interesting to speculate why the lower SIRT1 band is only present in young rat retinas, which also have the highest overall SIRT1 protein levels, as it may represent a more active form of SIRT1 that provides additional protection to the developing retinal neurons.

Interestingly, although SIRT1 decreased with age while SIRT2, SIRT6, and SIRT7 did not change significantly during aging in the rat retina, the mitochondrial Sirtuins (SIRT3, SIRT4, and SIRT5) were expressed maximally in adult retinas. This might be due to an increased demand for the antioxidant activities of the mitochondrial Sirtuins during adulthood in the retina, perhaps because of an enhanced reliance on mitochondrial oxidative phosphorylation at this life stage. The subsequent drop in expression of the mitochondrial Sirtuins in old rat retinas could also be partly responsible for age-related retinal dysfunction, and future studies should look to increase their expression in old rat retinas along with SIRT1 to determine which will have the most beneficial effects for delaying retinal neuronal functional decline.

To date, calorie restriction (CR) is the most well-known and accepted effective means to extend the lifespan without genetic or pharmacological intervention. The expression of all Sirtuins except SIRT4 increase as an effect of CR (Wątroba and Szukiewicz, 2016). SIRT6 has been found to play a role in the regulation of mammalian aging in mice (Grabowska et al., 2017). SIRT7 translocates from nucleoli to chromatin and the cytoplasm during replicative senescence (Grob et al., 2009). SIRT3 has also been studied extensively because it is the only mitochondrial Sirtuin that has been linked to longevity (Rose et al., 2003) One study has shown that SIRT3 is reduced in aging mouse haematopoietic stem cells (mHSCs), and SIRT3 overexpression in aged mHSCs enhanced their regenerative ability (Brown et al., 2013). However, few studies have compared the expression levels of Sirtuins among young, adult, and old animal tissues as in the current study. Investigation of Sirtuins expression in other animal tissues during development and aging may further shed light on their role in the aging process and provide avenues for therapeutic interventions for additional aging-related diseases.

## Conclusion

Our study has provided foundational knowledge about Sirtuins in the retinal neurons of humans, mice, and rats, which will be helpful for future investigations on retinal metabolism and Sirtuin regulation. The present study also provides information on specific Sirtuin family members that may be targeted pharmacologically to combat diseases of retinal neurons. Future studies on Sirtuins should examine their precise protective roles in retinal neurodegeneration and the mechanisms by which they mediate such protection to inform development of treatments for age-related retinal decline.

## Author Contributions

The study was conceived and designed by XZ and HL. Data collection and analyses were conducted by HL, MZ, KJ, JZ, WD, SF, TS, and XZ. The paper was written and revised by HL and XZ.

## Conflict of Interest Statement

The authors declare that the research was conducted in the absence of any commercial or financial relationships that could be construed as a potential conflict of interest. The reviewer SK and handling Editor declared their shared affiliation.

## References

[B1] Alcendor R. R. Gao S. Zhai P. Zablocki D. Holle E. Yu X. (2007). Sirt1 regulates aging and resistance to oxidative stress in the heart. *Circ. Res.* 100 1512–1521. 10.1161/01.RES.0000267723.65696.4a 17446436

[B2] Balaiya S. Ferguson L. R. Chalam K. V. (2012). Evaluation of sirtuin role in neuroprotection of retinal ganglion cells in hypoxia role of SIRT1 in sustaining RGC viability. *Invest. Ophthalmol. Vis. Sci.* 53 4315–4322. 10.1167/iovs.11-9259 22669716

[B3] Ban N. Ozawa Y. Inaba T. Miyake S. Watanabe M. Shinmura K. (2013). Light–dark condition regulates sirtuin mRNA levels in the retina. *Exp. Gerontol.* 48 1212–1217. 10.1016/j.exger.2013.04.010 23648587

[B4] Braidy N. Poljak A. Grant R. Jayasena T. Mansour H. Chan-Ling T. (2015). Differential expression of sirtuins in the aging rat brain. *Front. Cell. Neurosci.* 9:167 10.3389/fncel.2015.00167 PMC4424846 26005404

[B5] Brown K. Xie S. Qiu X. Mohrin M. Shin J. Liu Y. (2013). SIRT3 reverses aging-associated degeneration. *Cell Rep.* 3 319–327. 10.1016/j.celrep.2013.01.005 23375372 PMC3582834

[B6] Duan W. (2013). Sirtuins: from metabolic regulation to brain aging. *Front. Aging Neurosci.* 5:36. 10.3389/fnagi.2013.00036 23888142 PMC3719022

[B7] Geng Y. Wang J. Liang J. Xu C. Zhi Y. (2016). Expression of Sirt1 and Sirt2 in the injured optic retina of calorie–restricted rats. *Eye Sci.* 26 221–224. 10.3969/g.issn.1000-4432.2011.04.008 22187307

[B8] Grabowska W. Sikora E. Bielak-Zmijewska A. (2017). Sirtuins, a promising target in slowing down the ageing process. *Biogerontology* 18 447–476. 10.1007/s10522-017-9685-9 28258519 PMC5514220

[B9] Grob A. Roussel P. Wright J. E. McStay B. Hernandez-Verdun D. Sirri V. (2009). Involvement of SIRT7 in resumption of rDNA transcription at the exit from mitosis. *J. Cell Sci.* 122 489–498. 10.1242/jcs.042382 19174463 PMC2714433

[B10] Ho L. Titus A. S. Banerjee K. K. George S. Lin W. Deota S. (2013). SIRT4 regulates ATP homeostasis and mediates a retrograde signaling via AMPK. *Aging (Albany NY)* 5 835–849. 10.18632/aging.100616 24296486 PMC3868726

[B11] Jaliffa C. Ameqrane I. Dansault A. Leemput J. Vieira V. Lacassagne E. (2009). Sirt1 involvement in rd10 mouse retinal degeneration. *Invest. Ophthalmol. Vis. Sci.* 50 3562–3572. 10.1167/iovs.08-2817 19407027

[B12] Jeong H. Cohen D. E. Cui L. Supinski A. Savas J. N. Mazzulli J. R. (2012). Sirt1 mediates neuroprotection from mutant huntingtin by activation of the TORC1 and CREB transcriptional pathway. *Nat. Med.* 18 159–165. 10.1038/nm.2559 22179316 PMC3509213

[B13] Kim D. Nguyen M. D. Dobbin M. M. Fischer A. Sananbenesi F. Rodgers J. T. (2007). SIRT1 deacetylase protects against neurodegeneration in models for Alzheimer’s disease and amyotrophic lateral sclerosis. *EMBO J.* 26 3169–3179. 10.1038/sj.emboj.7601758 17581637 PMC1914106

[B14] Kim H. S. Xiao C. Wang R. H. Lahusen T. Xu X. Vassilopoulos A. (2010). Hepatic-specific disruption of SIRT6 in mice results in fatty liver formation due to enhanced glycolysis and triglyceride synthesis. *Cell Metab.* 12 224–236. 10.1016/j.cmet.2010.06.009 20816089 PMC2935915

[B15] Kim S. H. Park J. H. Kim Y. J. Park K. H. (2013). The neuroprotective effect of resveratrol on retinal ganglion cells after optic nerve transection. *Mol. Vis.* 19 1667–1676. 23901250 PMC3724955

[B16] Komlos D. Mann K. D. Zhuo Y. Ricupero C. L. Hart R. P. Liu A. Y. C. (2013). Glutamate dehydrogenase 1 and SIRT4 regulate glial development. *Glia* 61 394–408. 10.1002/glia.22442 23281078 PMC3552040

[B17] Liu B. Chen H. Johns T. G. Neufeld A. H. (2006). Epidermal growth factor receptor activation: an upstream signal for transition of quiescent astrocytes into reactive astrocytes after neural injury. *J. Neurosci.* 26 7532–7540. 10.1523/JNEUROSCI.1004-06.2006 16837601 PMC6674203

[B18] Mao X. B. You Z. P. Wu C. Huang J. (2017). Potential suppression of the high glucose and insulin-induced retinal neovascularization by sirtuin 3 in the human retinal endothelial cells. *Biochem. Biophys. Res. Commun.* 482 341–345. 10.1016/j.bbrc.2016.11.065 27856259

[B19] Mao Z. Hine C. Tian X. Van Meter M. Au M. Vaidya A. (2011). SIRT6 promotes DNA repair under stress by activating PARP1. *Science* 332 1443–1446. 10.1126/science.1202723 21680843 PMC5472447

[B20] Mimura T. Kaji Y. Noma H. Funatsu H. Okamoto S. (2013). The role of SIRT1 in ocular aging. *Exp. Eye Res.* 116 17–26. 10.1016/j.exer.2013.07.017 23892278

[B21] Morris B. J. (2013). Seven sirtuins for seven deadly diseases of aging. *Free Radic. Biol. Med.* 56 133–171. 10.1016/j.freeradbiomed.2012.10.525 23104101

[B22] Mortuza R. Feng B. Chakrabarti S. (2015). SIRT1 reduction causes renal and retinal injury in diabetes through endothelin 1 and transforming growth factor β1. *J. Cell Mol. Med.* 19 1857–1867. 10.1111/jcmm.12557 25753689 PMC4549036

[B23] Niven J. E. Laughlin S. B. (2008). Energy limitation as a selective pressure on the evolution of sensory systems. *J. Exp. Biol.* 211 1792–1804. 10.1242/jeb.017574 18490395

[B24] Ozawa Y. Kubota S. Narimatsu T. Yuki K. Koto T. Sasaki M. (2010). Retinal aging and sirtuins. *Ophthalmic Res.* 44 199–203. 10.1159/000316484 20829644

[B25] Peng C. H. Chang Y. L. Kao C. L. Tseng L. M. Wu C. C. Chen Y. C. (2010). SirT1—a sensor for monitoring self-renewal and aging process in retinal stem cells. *Sensors* 10 6172–6194. 10.3390/s100606172 22219708 PMC3247753

[B26] Pennington K. L. DeAngelis M. M. (2016). Epigenetic mechanisms of the aging human retina. *J Exp Neurosci.* 9(Suppl. 2) 51–79. 10.4137/JEN.S25513 26966390 PMC4777243

[B27] Rose G. Dato S. Altomare K. Bellizzi D. Garasto S. Greco V. (2003). Variability of the *SIRT3* gene, human silent information regulator *Sir2* homologue, and survivorship in the elderly. *Exp. Gerontol.* 38 1065–1070. 10.1016/S0531-5565(03)00209-2 14580859

[B28] Sasaki M. Yuki K. Kurihara T. Miyake S. Noda K. Kobayashi S. (2012). Biological role of lutein in the light-induced retinal degeneration. *J. Nutr. Biochem.* 23 423–429. 10.1016/j.jnutbio.2011.01.006 21658930

[B29] Satoh A. Stein L. Imai S. (2011). “The role of mammalian sirtuins in the regulation of metabolism, aging, and longevity,” in *Histone Deacetylases: the Biology and Clinical Implication* eds Yao T. P. Seto E. (Berlin: Springer) 126–163. 10.1007/978-3-642-21631-2_7 PMC3745303 21879449

[B30] Shindler K. S. Ventura E. Rex T. S. Elliott P. Rostami A. (2007). SIRT1 activation confers neuroprotection in experimental optic neuritis. *Invest. Ophthalmol. Vis. Sci.* 48 3602–3609. 10.1167/iovs.07-0131 17652729 PMC1964753

[B31] Shulyakova N. Sidorova-Darmos E. Fong J. Zhang G. Mills L. R. Eubanks J. H. (2014). Over-expression of the Sirt3 sirtuin protects neuronally differentiated PC12 Cells from degeneration induced by oxidative stress and trophic withdrawal. *Brain Res.* 1587 40–53. 10.1016/j.brainres.2014.08.066 25194924

[B32] Sidorova-Darmos E. Wither R. G. Shulyakova N. Fisher C. Ratnam M. Aarts M. (2014). Differential expression of sirtuin family members in the developing, adult, and aged rat brain. *Front. Aging Neurosci.* 6:333. 10.3389/fnagi.2014.00333 25566066 PMC4270178

[B33] Silberman D. M. Ross K. Sande P. H. Kubota S. Ramaswamy S. Apte R. S. (2014). SIRT6 is required for normal retinal function. *PLOS ONE* 9:e98831. 10.1371/journal.pone.0098831 24896097 PMC4045872

[B34] Someya S. Yu W. Hallows W. C. Xu J. Vann J. M. Leeuwenburgh C. (2010). Sirt3 mediates reduction of oxidative damage and prevention of age-related hearing loss under caloric restriction. *Cell* 143 802–812. 10.1016/j.cell.2010.10.002 21094524 PMC3018849

[B35] Wątroba M. Szukiewicz D. (2016). The role of sirtuins in aging and age-related diseases. *Adv. Med. Sci.* 61 52–62. 10.1016/j.advms.2015.09.003 26521204

[B36] Wu Y. Li X. Zhu J. X. Xie W. Le W. Fan Z. (2011). Resveratrol-activated AMPK/SIRT1/autophagy in cellular models of Parkinson’s disease. *Neurosignals* 19 163–174. 10.1159/000328516 21778691 PMC3699815

[B37] Zeng Y. Yang K. (2015). Sirtuin 1 participates in the process of age-related retinal degeneration. *Biochem. Biophys. Res. Commun.* 468 167–172. 10.1016/j.bbrc.2015.10.139 26522222

[B38] Zheng T. Lu Y. (2011). Changes in SIRT1 expression and its downstream pathways in age-related cataract in humans. *Curr. Eye Res.* 36 449–455. 10.3109/02713683.2011.559301 21501079

[B39] Zuo L. Khan R. S. Lee V. Dine K. Wu W. Shindler K. S. (2013). SIRT1 promotes RGC survival and delays loss of function following optic nerve crush. *Invest. Ophthalmol. Vis. Sci.* 54 5097–5102. 10.1167/iovs.13-12157 23821198 PMC3726244

